# Eating disorders and the incel community

**DOI:** 10.3389/fpsyg.2026.1940859

**Published:** 2026-09-04

**Authors:** Siobha Murphy, Darren William Roddy

**Affiliations:** Department of Psychiatry, Royal College of Surgeons in Ireland, Dublin, Ireland

**Keywords:** body dysmorphic disorder (BDD), eating disorders, incel, masculinity, online subculture, red pill ideology

## 1 Introduction

Much discussion has arisen regarding the root causes and larger implications of the ever-growing global masculinity crisis (Rottweiler et al., 2024). Male extremism and violence against women has become increasingly normalized (Akudolu et al., 2023). This is largely thought to be the consequence of the “manosphere,” a group of online masculinist subcultures indoctrinating followers with misogynistic ideology (U N Women, 2025).

The scale at which manosphere content has permeated the online lives of men cannot be understated; according to the Movember Foundation, two-thirds of young men regularly engage with masculinity influencers online (Movember.com, 2025). Additionally, due to its inflammatory nature and high levels of engagement, the content is heavily pushed by algorithms. This has created a radicalizing movement that has heavily influenced the psychosocial development of a generation of young men (Regehr et al., 2025). Of these, those most attracted are from vulnerable demographics (Botto and Gottzén, 2023), including those that have been bullied, abused (Childrenssociety.org.uk, 2023) or have conditions such as poor self-esteem, anxiety, depression and autism spectrum disorder (ASD) (Costello et al., 2025).

Rather than seeking out mental health services, which is seen as “weak” or emasculating behavior within current patriarchal society (Staiger et al., 2020), young men follow rigid, hyperbolic guidelines for masculinity established by these groups to try and achieve self-actualisation (O'Rourke et al., 2024). Many of these groups' definition of masculinity can only be achieved via toxic, often self-destructive behavior (Murphy, 2023). The “masculine” bodytype, as dictated by these groups, is tall, muscular and lean. This build is unachievable for most, or only through excessive exercise, dieting and/or drug use.

The author argues that one of these subcultures in particular—incels—has large numbers of members who could potentially meet the diagnostic criteria for eating disorders (ED), and exhibit signs of body dysmorphic disorder (BDD). Additionally, previous study has shown that there are markedly high rates of common ED and BDD comorbidities in the incel community (Hambleton et al., 2022). This could be a topic of growing interest, as the subculture has evolved in specific ways that actively encourages this effect on followers (Sousbois, 2025). The prevalence of these conditions in incels has yet to be documented, and could reveal new insights into the evolution of ED and BDD presentation and demographics in the 21st century.

## 2 What are incels?

“Incels” or “involuntary celibates” are a predominantly male and heterosexual online subculture where men blame and objectify women in retaliation for their inability to procure a romantic/sexual partner (Aiolfi et al., 2024). Within it, patriarchy is thought to be a conspiracy created by women to cover up their covert dominance over men in the social hierarchy (Sparks et al., 2022). They segregate society by sex, in which people are ranked based on physical appearance (O'Malley and Helm, 2022). There is a belief that men are entitled to sex, but are continuously denied by women based solely on their appearance (Gosse et al., 2024). In its extremist form, inceldom promotes rape and assault, and has been linked to acts of mass violence (Meleagrou-Hitchens and Hughes, 2026).

Incels use the term “red pill” to describe the belief in their “realization” that men do not hold systemic power, and are in fact a subjugated underclass. By “taking the red pill” (a reference to the 1999 film *The Matrix*) you have declared your belief in and desire for a return to an explicitly patriarchal society (Botto and Gottzén, 2023). Red pill ideology often focuses on individual-level attempts to climb the social hierarchy via the acquisition of improved physical appearance with the goal of a heterosexual relationship (Davis, 2022). This is achieved through trending efforts encouraged by figureheads in the community such as “looksmaxxing”—a growing practice involving obsessive dietary restriction, skin care, hair care, exercise and other potentially extreme routines to enhance physical attractiveness (Farrell, 2024). Gym/fitness content is often the “cultural touchpoint” used to connect with and indoctrinate young men into incel ideology. By engaging with this, they are soon funneled into masculinity status content. This then leads to rapidly consuming degrading health content, including health-risk taking behaviors, hate speech, explicitly misogyny and violence (Movember.com., 2026).

The traditional, rigid societal roles and standards offered by the rhetoric of the community soothe feelings of helplessness while simultaneously cultivating problematic behaviors (Murphy, 2023). Much in the same way that patriarchal society encourages BDD and ED behaviors in women (“heroin chic” in the 1990s and 2000s [Denham, 2008), the rise of Ozempic in the 2020s (Muhammad and Ahmed, 2025)], these standards also encourage harmful body image and behaviors in men.

## 3 Eating disorders, body dysmorphic disorder and the red pill

EDs are a group of prevalent psychiatric illnesses characterized by persistent disordered eating and/or eating-related behaviors leading to impaired food consumption (Fernández-Aranda et al., 2023). They are severe conditions with the potential to lead to significantly impaired physical and psychosocial health (National Institute of Mental Health, 2024). Both the International Classification of Diseases 11 (ICD-11) and Diagnostic and Statistical Manual 5 (DSM-5) name 6 main EDs. The three most well-known are Anorexia Nervosa (AN), Bulimia Nervosa (BN) and Binge Eating Disorder (BED). Three more, mainly regarded as childhood disorders have been added: Avoidant-Restrictive Food Intake Disorder (ARFID), Pica and Rumination Disorder (Claudino et al., 2019) (Mancuso et al., 2015). An emerging yet controversial ED is orthorexia. Characterized by an obsessive preoccupation with dietary purity, its classification remains a subject of debate within the mental health and psychiatric community (Horovitz and Argyrides, 2023).

BDD is classified as an obsessive-compulsive related disorder in the DSM-5. To meet the diagnostic criteria, the patient must be preoccupied with one or more minor or non-existent flaws in their appearance for collectively 1 h or more a day. The supposed appearance flaws cause repetitive, compulsive behaviors such as reassurance checking, mirror checking, or compulsively comparing one's physical appearance with others (Ruck et al., 2024). People with Muscle Dysmorphia BDD have a preoccupation with their muscle mass or build being too small (Foster et al., 2015). Compared to other forms, those with Muscle Dysmorphia BDD have even higher rates of substance abuse and suicidality, and are more likely to use anabolic steroids (Pope et al., 2005).

A hallmark of red pill ideology, as peddled by incels, is that physique is the key to ascending the social hierarchy (Vallerga and Zurbriggen, 2023). Alongside mental health concerns being dismissed as weakness and therapy as emasculating, supplementing healthcare with extreme diet and exercise is encouraged (Botto and Gottzén, 2023). Much like other individualist strategies attempted to counter crisis conditions under neoliberal capitalism, agency and power are said to be found in discipline and expensive “wellness” rituals and products (Bujalka et al., 2026). This mirrors the psychology underlying the development of EDs and BDD, both of which can start as a bid for control of the self in an unstable internal or external environment (Froreich et al., 2016).

Many figureheads in the community have exhibited concerning relationships with food. Andrew Tate was quoted on X in 2023 saying “Food's awful and eating sucks. I hate eating. I hate feeling full…. Imagine how stupid you have to be to find food entertaining. Literally embarrassing” (Williams, 2023). Jordan Peterson once spent months consuming nothing but water, beef and salt, ending up in a coma in 2019. Responding to concerns that this may have been linked to his diet, he denied any potential relationship, instead claiming that it was due to his clonazepam dependence (Wilkins, 2025). Such performative disgust and restrictive dieting is reminiscent of an ED.

As a result of stratified socialization, men and women exhibit different compensatory ED behaviors. Men are more likely to hide theirs with intense sporting practices and strict dietary regimes, and are more likely to have concurrent Muscle Dysmorphia BDD (Capuano et al., 2025). Anabolic steroid use, commonly seen in people with Muscle Dysmorphia BDD, is becoming increasingly prevalent in young men who identify as incels (Cao, 2025).

The prevalence of EDs in men has increased rapidly over the past 25 years (Liu et al., 2025). In the early 2000s, 10% of reported clinical ED cases occur in men, but a 2022 report found that the number is now closer to 25%. However, this is still thought to be a gross underestimation of the true number of cases (Gorrell and Murray, 2019). While obviously not the sole cause, this matches the timeline of the birth and rise of the incel community.

Despite modern consensus that EDs are not exclusive to women, men are collectively less likely to be assessed and diagnosed. The barriers are both individual and sociological, suppressing and emasculating men who discuss their struggles with disordered self-image and eating (Lehe et al., 2025). The documented numbers not reflecting reality could indicate numbers of young men seeking support in inappropriate, non-clinical environments, including incel community online. Additionally, men may present with symptoms that closer align with ED presentations such as orthorexia and exercise addiction (Capuano et al., 2025) (Wachten and Strahler, 2026), which are still viewed as sub-clinical diagnoses or have yet to be formally classified (Horovitz and Argyrides, 2023).

While these men would not self-describe as having eating disorders or body dysmorphia, their collective behavior and thought processes align with the diagnostic criteria for these illnesses. There is undoubtedly more nuance on an individual level, but it could be proposed that young men with these illnesses are uniquely susceptible to the communities that prop themselves up as a socially acceptable alternative to mental health resources.

## 4 The new face of the pathology: looksmaxxing and its consequences

While the beliefs of previously established figureheads are concerning, it is the new faces of the incel community and the wider manosphere that threaten large-scale perpetuation of these behaviors. In recent years, the agenda has shifted from one of purely anti-feminism to that of dominant “alpha masculinity,” prioritizing male self-help and wellbeing (Bujalka et al., 2026).

“Looksmaxxing,” a term rooted in the incel community, describes a practice involving obsessive dietary restriction, skin care, hair care, exercise and other extreme routines to enhance physical attractiveness. In the 20^th^ and early 21st century, cultivating one's physical appearance to this extent went against behaviors that were seen as “masculine” in the Western world. This wider cultural shift speaks to how gender—in this case, masculinity—is but a performance that changes throughout history. In the age of social media, we have arrived at the “red pill” version, which has had unintended ill-effects on the men who have sought these spaces as a way of validating their masculinity. A study from Dalhousie University found that the looksmaxx community “subject users to masculine demoralization, wherein they are seen as failed men and encouraged to self-harm” (Halpin et al., 2025).

These men are taught that in order to be valued, they must undergo intensive “self-improvement” regimes. These include techniques such as “mewing” (an unproven orthotropic technique to alter the jawline and face shape by flattening the tongue against the roof of the mouth) (Medex, 2025), restrictive dieting, intense physical exercise, buccal fat removal, Botox, filler, injecting anabolic steroids, and limb-lengthening surgery. Something notable about the effects of “looksmaxxing” is the obsessive nature of the health behaviors exhibited by members. This, along with the functional impacts of the procedures speaks to the potential psychiatric conditions present in the looksmaxxing community, particularly EDs and BDD (Murphy, 2023).

An influencer linked with propagating looksmaxxing is Braden Peters, known online as “Clavicular” (Okundaye, 2026). He is largely controversial due to his advocacy of facial “bonesmashing” (taking a hammer to his jaw, breaking the mandible to reshape it), anabolic steroid use and crystal meth consumption as an appetite suppressant (Henderson, 2026). Clavicular has suffered ill-health from the interventions taken to alter his appearance. At 14, he began injecting himself with testosterone supplements while at home over the Covid-19 pandemic (Mack, 2026). In 2025, he stated that this has left him infertile (Page, 2026). The obsessive nature of his search for an “optimized” physical appearance has people speculating whether he has BDD (Okundaye, 2026).

## 5 Comparing ED comorbidities in Incels and the general populus

Attraction to and presence in the incel community is thought to stem from two sources; antisocial personality traits like Machiavellianism, psychopathy and narcissism (Akdag and Blakey, 2025), or from a history of being abused and bullied, low self-esteem and having high rates of autistic traits compared to the rest of the population (Costello et al., 2025). While there has been some research into a link between personality disorders such as Borderline Personality Disorder and EDs (Gabriel and Waller, 2014), the latter poses the stronger argument for a relationship by being common ED comorbidities (Juli et al., 2023).

The incel community has high rates of various mental health conditions and ASD compared to the general population (Tirkkonen and Vespermann, 2023). An online survey by the International Center for the Study of Violent Extremism found that of the 272 incels questioned, 64% reported depressive symptoms, 48% reported suicidal ideation, 60% reported anxiety symptoms and 28% reported symptoms of Post-Traumatic Stress Disorder (PTSD) (Speckhard et al., 2021). Almost one-quarter of participants reported ASD symptoms, a statistic that has been replicated in other studies (Costello et al., 2025).

This is in stark contrast to global statistics, where depression affects 14.6% of the population in high income countries (Lim et al., 2018) and 5.3% of the global populus report suicidal ideation (Singichetti et al., 2025). Up to 33.7% of people report anxiety symptoms (Kaczkurkin and Foa, 2015) and an estimated 3.9% of people globally report symptoms of PTSD (World Health Organization, 2024). The global prevalence of ASD is 0.6% (Salari et al., 2022).

The rates of these conditions in people with EDs are comparable to that of them in incels. 51.5% of adolescents with AN will have a concurrent depressive disorder. (Zhang et al., 2020) 23% of adults with AN report lifetime suicidal ideation (Smith et al., 2018). The most common psychiatric comorbidities are anxiety disorders, with up to 62% of people with AN reporting symptoms (Ulfvebrand et al., 2015). Between 16.1–22.7% of AN patients, 32.4–66.2% of BN patients and 24.02–31.6% of BED patients experience PTSD symptoms (Mitchell et al., 2021). The average prevalence of ASD with EDs is 22.9% (Huke et al., 2013). These markedly high numbers are comparable with the statistics of such conditions present in the incel population. The high rates of ED comorbidities could speak to a shared vulnerability to EDs and/or BDD.

## 6 Conclusion and suggestions for future research

The author argues that the above information suggests that red pill ideology, as perpetrated by incels, encourage EDs/BDD in its believers, and exploits the current presence of these illnesses in followers. Behaviors linked with these diseases are encouraged and celebrated as part of achieving a specific desired performance of “manhood.” In interrogating the links between red pill ideology and EDs, the dangers of the incel community on body image and resulting eating/exercise habits become apparent. Additionally, there are markedly high numbers of known ED/BDD comorbidities present in the incel community, which could be indicative of the group's vulnerability to disordered eating and body image. Further researching and quantifying this phenomenon could improve our understanding of EDs and BDD in men in the 21st century. This could help parse whether this occurs as a consequence of red pill ideology, whether an ED/BDD makes one more susceptible to the movement, or a combination of the two.
